# Sodium bicarbonate in the prevention of cardiac surgery-associated acute kidney injury: a systematic review and meta-analysis

**DOI:** 10.1186/s13054-014-0517-x

**Published:** 2014-09-12

**Authors:** Hong-Tao Tie, Ming-Zhu Luo, Ming-Jing Luo, Min Zhang, Qing-Chen Wu, Jing-Yuan Wan

**Affiliations:** Department of Cardiothoracic Surgery, The First Affiliated Hospital of Chongqing Medical University, Chongqing, 400016 China; Chongqing Key Laboratory of Biochemistry and Molecular Pharmacology, Chongqing Medical University, Chongqing, 400016 China; The Children’s Hospital of Chongqing Medical University, Chongqing, 400016 China

## Abstract

**Introduction:**

Sodium bicarbonate (SBIC) was reported to be a promising approach to prevent cardiac surgery-associated acute kidney injury (CSA-AKI). However, the results remain controversial. We conducted a systematic review and meta-analysis to evaluate the efficacy and safety of SBIC on the prevention of CSA-AKI in adult patients undergoing cardiac surgery.

**Methods:**

PubMed, EMbase, Web of science, EBSCO, and Cochrane library databases were systematically searched. Randomized controlled trials (RCTs) assessing the effect of SBIC versus placebo on the prevention of CSA-AKI in adult patients undergoing cardiac surgery were included. Two investigators independently searched articles, extracted data, and assessed the quality of included studies. The primary outcome was the incidence of CSA-AKI. Meta-analysis was performed using random-effects models.

**Results:**

Five RCTs involving 1079 patients were included in the meta-analysis. Overall, compared with placebo, SBIC was not associated with a reduced risk of CSA-AKI (relative risk [RR] 0.99; 95% confidence interval [CI] 0.78 to 1.24; *P* = 0.911). SBIC failed to alter the clinical outcomes of hospital length of stay (weighted mean difference [WMD] 0.23 days; 95%CI −0.88 to 1.33 days; *P* = 0.688), renal replacement therapy (RR 0.94; 95%CI 0.49 to 1.82; *P* = 0.861), hospital mortality (RR 1.37; 95%CI 0.46 to 4.13; *P* = 0.572), postoperative atrial fibrillation (RR 1.02; 95%CI 0.65 to 1.61; *P* = 0.915). However, SBIC was associated with significant increased risks in longer duration of ventilation (WMD 0.64 hours; 95%CI 0.16 to 1.11 hours; *P* = 0.008), longer ICU length of stay (WMD 2.06 days; 95%CI 0.54 to 3.58 days; *P* = 0.008), and increased incidence of alkalemia (RR 2.21; 95%CI 1.42 to 3.42; *P* <0.001).

**Conclusions:**

SBIC could not reduce the incidence of CSA-AKI. Contrarily, SBIC prolongs the duration of ventilation and ICU length of stay, and increases the risk of alkalemia. Thus, SBIC should not be recommended for the prevention of CSA-AKI and perioperative SBIC infusion should be administrated with caution.

**Electronic supplementary material:**

The online version of this article (doi:10.1186/s13054-014-0517-x) contains supplementary material, which is available to authorized users.

## Introduction

Acute kidney injury (AKI) is a frequent and severe postoperative complication in patients undergoing cardiac surgery [1] with an incidence varying from 36.3 to 52.0% [2–6]. With increasing interest, this topic has been specifically referred to as cardiac surgery-associated acute kidney injury (CSA-AKI). CSA-AKI could contribute to increased in-hospital mortality, 5-year mortality, 30-day readmission, requirement for renal replacement therapy (RRT), ICU length of stay, and total postoperative cost [7–13]. Considering the poor prognosis and increasing medical cost, prophylaxis of CSA-AKI is urgently needed. Although many strategies have tried to reduce the incidence of CSA-AKI [14,15], effective methods to prevent CSA-AKI unfortunately remain to be established due to underpowered evidence and controversial conclusions.

The pathogenesis of CSA-AKI is multifactorial, including ischemia and reperfusion injury, inflammation, oxygen free radicals, oxidative stress, and free hemoglobin [7,16]. An experimental study demonstrated that urinary alkalinization with sodium bicarbonate (SBIC) could prevent oxidant injury to the kidney by eliminating oxygen species [17]. Accordingly, a randomized double-blind trial involving 100 patients suggested that intravenous SBIC could also effectively reduce the incidence of AKI in patients undergoing on-pump cardiac surgery [3]. In contrast to this promising finding, however, accumulating relevant randomized controlled trials (RCTs) showed that intravenous SBIC failed to improve renal function or prevent CSA-AKI [2,4–6]. Moreover, one of these trials found that intravenous SBIC might increase mortality [4]. Considering these inconsistent effects and even potential harms, we therefore conducted a systematic review and meta-analysis of RCTs to evaluate the efficacy and safety of SBIC on the prevention of CSA-AKI in adult patients undergoing cardiac surgery.

## Materials and methods

This systematic review and meta-analysis were conducted according to the guidance of the Preferred Reporting Items for Systematic Reviews and Meta-analysis statement [18] and the *Cochrane Handbook for Systematic Reviews of Interventions* [19]. All analyses are based on previous published studies, thus no ethical approval and patient consent are required.

### Literature search and selection criteria

PubMed, EMbase, Web of science, EBSCO, and the Cochrane library were systematically searched from inception to 15 March 2014, with the following keywords: sodium bicarbonate, acute kidney injury, and cardiac surgery. No limitation was enhanced. To include additional eligible studies, the reference lists of retrieved studies and relevant reviews were also hand-searched and the process above was performed repeatedly until no further article was identified. Conference abstracts meeting the inclusion criteria were also included.

The inclusion criteria were as follows: study population, adult patients undergoing cardiac surgery; intervention, SBIC; control, placebo; outcome measure, incidence of CSA-AKI; and study design, RCT.

### Data extraction and outcome measures

The following information was extracted for the included RCTs: first author, publication year, sample size, baseline characteristics of patients, surgery type, intervention of SBIC, intervention of control, study design, definition of CSA-AKI, incidence of CSA-AKI, duration of ventilation, ICU length of stay, hospital length of stay (HLOS), hospital mortality, incidence of RRT, incidence of postoperative atrial fibrillation (POAF), and incidence of alkalemia. The author would be contacted to acquire the data when necessary.

The primary outcome was the incidence of CSA-AKI. Secondary outcomes included the duration of ventilation, ICU length of stay, HLOS, hospital mortality, incidence of RRT, incidence of POAF, and incidence of alkalemia.

### Assessment for risk of bias

The risk of bias tool was used to assess the quality of individual studies in accordance with the *Cochrane Handbook for Systematic Reviews of Interventions* [19], and the following sources of bias were considered: selection bias, performance bias, attrition bias, detection bias, reporting bias, and other potential sources of bias. The overall risk of bias for each study was evaluated and rated: low, when the risk of bias was low in all key domains; unclear, when the risk of bias was low or unclear in all key domains; and high, when the risk of bias was high in one or more key domains [20]. Two investigators independently searched articles, extracted data, and assessed the quality of included studies. Any discrepancy was solved by consensus.

### Statistical analysis

The relative risks (RRs) with 95% confidence intervals (CIs) for dichotomous outcomes (incidence of RRT, hospital mortality, incidence of alkalemia, and incidence of POAF) and weighted mean differences (WMDs) with 95% CIs for continuous outcomes (duration of ventilation, ICU length of stay, and HLOS) were used to estimate the pooled effects. For continuous outcomes expressed as medians with ranges, the means and relevant variances were estimated using an elementary inequality and approximation [21]. All meta-analyses were performed using random-effects models with DerSimonian and Laird weights. Heterogeneity was assessed with the *I*^2^ statistic, which quantified the proportion of total variation caused by heterogeneity instead of chance. The heterogeneity was perceived as low (*I*^2^*s*tatistic between 25 and 50%), moderate (*I*^2^ statistic between 50 and 75%), and high (*I*^2^*s*tatistic >75%) [22]. Sensitivity analysis was performed to detect the influence of a single study on the overall estimate via omitting one study in turn and pooling the remaining ones. Additionally, sensitivity analyses according to various exclusion criteria (full-text article, large-scale trials (sample size >100), patients with pre-existing renal impairment or chronic kidney disease, elective or urgent surgery, and only elective surgery) were also conducted. Owing to the limited number (<10) of included studies, publication bias was not assessed. *P* <0.05 in two-tailed tests was considered statistically significant. All statistical analyses were performed with Stata 12.0 software (StataCorp, College Station, TX, USA), except for the risk of bias that was conducted using Review Manager Version 5.1 (The Cochrane Collaboration, Software Update, Oxford, UK).

## Results

### Literature search and study characteristics

**Figure 1:**
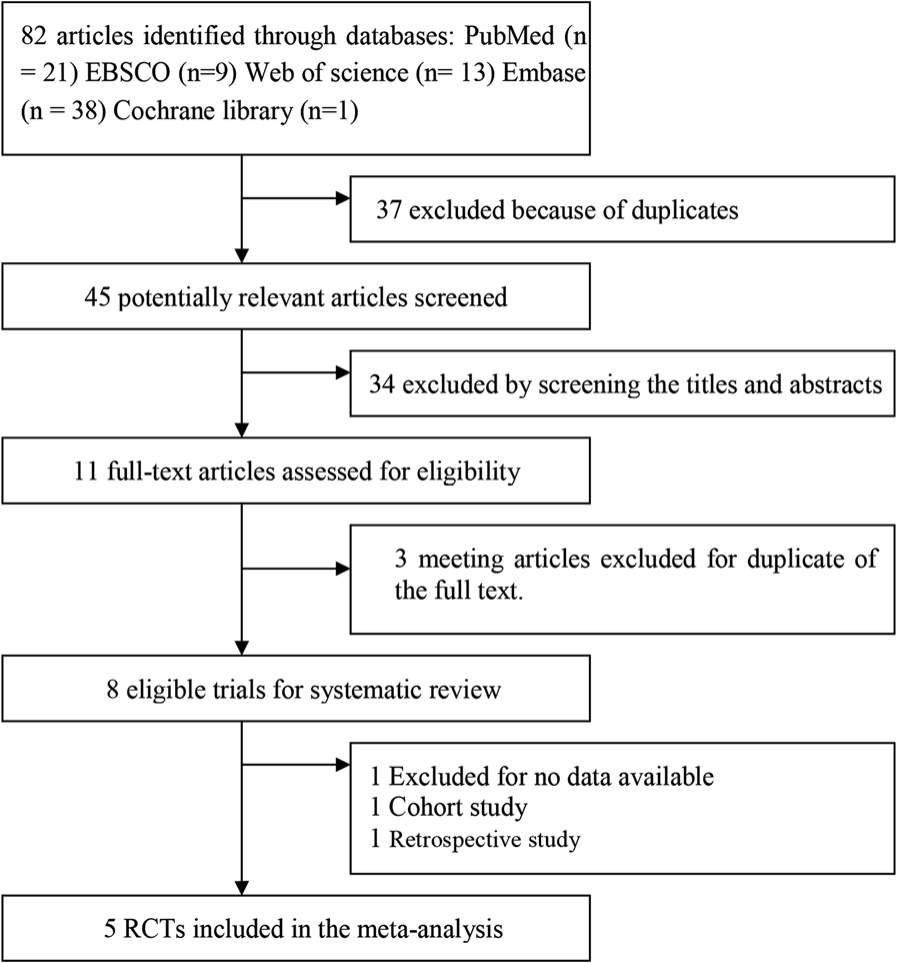
**Flow chart for the systematic review and meta-analysis.** RCT, randomized controlled trial.

The flow chart for the selection process and detailed identification is presented in Figure 1. Eighty-two publications were identified through the initial search of databases. Ultimately, five RCTs were included in the meta-analysis [2–6].

The baseline characteristics of the five eligible RCTs in the meta-analysis are summarized in Table 1, and the outcomes are presented in Additional file 1. The five studies were published between 2009 and 2013, and sample sizes ranged from 92 to 427 with a total of 1,079. The participants in all trials were adults (>18 years). Of these five RCTs, three studies enrolled patients with pre-existing renal impairment [2,4,5], one study enrolled those at the risk of postoperative acute renal dysfunction [3], and one study enrolled patients at risk of AKI or with pre-existing renal impairment [6]. Cardiopulmonary bypass was used in cardiac operation in all of the trials. The surgery types in three studies were elective or urgent surgery [3–5], while the other two studies only involved elective surgery [2,6]. Four studies administrated SBIC via intravenous infusion with a procedure of bolus injection and continuous infusion [3–6], and the remaining trial infused SBIC intravenously with a procedure of only continuous infusion [2]. Four studies defined CSA-AKI as an increase in serum creatinine (sCr) >25% or 0.5 mg/dl from baseline to peak value at any time within the first 5 postoperative days [2,3,5,6], while the other trial was according to Acute Kidney Injury Network classification [4]. Among the five RCTs, all studies reported the outcome of CSA-AKI, four studies reported the duration of ventilation, ICU length of stay, HLOS, and hospital mortality [3–6], three studies reported the incidence of POAF and the incidence of alkalemia [3–5], and three studies reported the incidence of RRT [3,4,6].

**Table 1:** **Main characteristics of the five RCTs included in the meta-analysis**

**Study ID**	**Number of patients (SBIC/control)**	**Population**	**Surgery type**	**Intervention of SBIC**	**Definition of CSA-AKI**
Haase and colleagues [3]	50/50	>18 years/at risk of postoperative acute renal dysfunction	Elective or urgent cardiac surgery; CPB	SBIC: 0.5 mmol/kg BW (bolus) 1 hour after the induction of anesthesia; continuous infusion of 0.15 mmol/kg/hour over 23 hours, IV	sCr criteria^a^ within the first 5 days after CPB
Del Duca and colleagues [2]	55/55	>18 years/ stable CKD	Nonemergency cardiac surgery; CPB	SBIC: 150 mEq, at 1 ml/hour for 6 hours prior to CPB, IV	sCr criteria within the first 5 days after CPB
Haase and colleagues [4]	174/176	>18 years/pre-existing renal impairment	Elective or urgent cardiac surgery; CPB	SBIC: 0.5 mmol/kg BW (bolus) 1 hour after the induction of anesthesia; continuous infusion of 0.2 mmol/kg/hour over 23 hours, IV	Acute Kidney Injury Network criteria^b^ within 5 days after surgery
Kristeller and colleagues [5]	44/48	>18 years/ stage 3 or higher CKD or GFR <60 ml/minute/1.73 m^2^	Elective or urgent cardiac surgery; CPB	SBIC: 150 mEq, at 3 ml/kg/hour from 1 hour preoperatively to the patients starting on CPB, IV; continuous infusion of 1 ml/kg/hour during and for 6 hours after CPB, IV	sCr criteria within the first 5 postoperative days
McGuinness and colleagues [6]	215/212	>18 years/ at risk of AKI or pre-existing renal impairment	Elective cardiac surgery; CPB	SBIC: 0.5 mmol/kg BW (=bolus) over 1 hour after the induction of anesthesia; continuous infusion of 0.2 mmol/kg/hour over 23 hours, IV	sCr criteria within the first 5 postoperative days

AKI, acute kidney injury; BW, body weight; CKD, chronic kidney disease; CPB, cardiopulmonary bypass; CSA-AKI, cardiac surgery-associated acute kidney injury; GFR, glomerular filtration rate; IV, intravenous injection; RCT, randomized controlled trial; SBIC, sodium bicarbonate; sCr, serum creatinine. ^a^sCr criteria: an increase in sCr >25% or 0.5 mg/dl from baseline to peak value at any time. ^b^Acute Kidney Injury Network criteria: an absolute increase in sCr ≥0.3 mg/dl (26.4 μmol/l) or 50% (1.5-fold from baseline)/a reduction in urine output (<0.5 ml/kg per hour for more than 6 hours).

### Assessment of risk of bias

**Figure 2:**
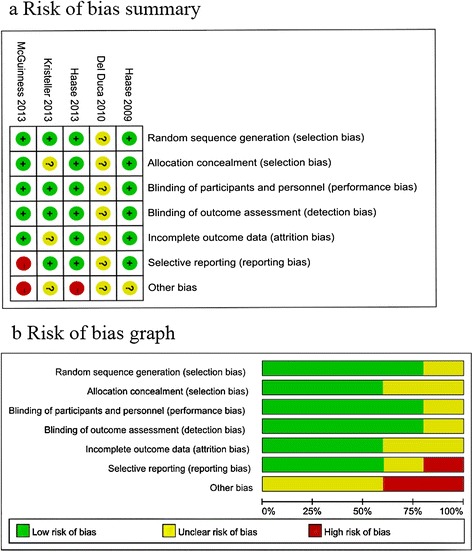
**Risk of bias assessment. (a)** Authors' judgments about each risk of bias item for each included study [2–6]. **(b)** Authors' judgments about each risk of bias item presented as percentages across all included studies.

Risk of bias analysis (Figure 2) showed that two studies had high risk of bias because they were terminated ahead of schedule [4,6], and one of these also had reporting bias since it did not report the adverse events and safety concerns as mentioned in their protocol [6].

### Primary outcome: incidence of cardiac surgery-associated acute kidney injury

**Figure 3:**
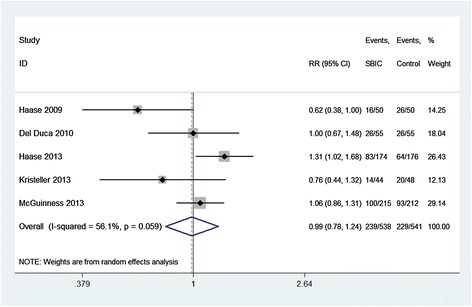
**Forest plot for the meta-analysis of the incidence of cardiac surgery-associated acute kidney injury.** Five randomized controlled trials included [2–6]. CI, confidence interval; RR, relative risk; SBIC, sodium bicarbonate.

All five studies reported the outcome of CSA-AKI and all of these were involved to estimate the pooled effect of SBIC on the prevention of CSA-AKI. Overall, the incidence of the CSA-AKI was 44.4% and 42.3% in the SBIC and control groups, respectively. Analyzed with a random-effects model, the pooled estimate of the five RCTs suggested that SBIC was not associated with a significant reduction in the incidence of CSA-AKI (RR = 0.99; 95% CI = 0.78 to 1.24; *P* = 0.911), with a moderate heterogeneity among the studies (*I*^2^ = 56.1%, heterogeneity *P* = 0.059) (Figure 3).

### Sensitivity analysis

Since moderate heterogeneity was observed among the studies for the CSA-AKI, sensitivity analysis by omitting one study in each turn was performed to detect the source of heterogeneity and the stability of the pooled estimate. Although the source of the heterogeneity was not found, the results showed that no single study could substantially alter the pooled estimates with RRs ranging from 0.90 (95% CI = 0.71 to 1.15) to 1.09 (95% CI = 0.92 to 1.30). Additionally, sensitivity analyses according to various exclusion criteria suggested that the pooled estimates remained not statistically significant, as shown in Table 2.

**Table 2:** **Sensitivity analysis according to various exclusion criteria for CSA-AKI**

**Criteria**	**Number of trials**	**Number of patients**	**SBIC group**	**Control group**	**RR (95% CI)**	***P*** **value**	***I*** ^**2**^ **(%)**
All included trials [2–6]	5	1,079	239/538	229/541	0.99 (0.78 to 1.24)	0.911	56.1
Full-text trials [3–6]	4	969	213/483	203/486	0.97 (0.72 to 1.29)	0.824	66.8
Large-scale trials (sample size >100) [2–4,6]	4	987	225/494	209/493	1.02 (0.80 to 1.31)	0.867	61
Patients with pre-existing renal impairment or CKD [2,4,5]	3	552	123/273	110/279	1.08 (0.80 to 1.45)	0.631	45.9
Elective or urgent surgery [3–5]	3	542	113/268	110/274	0.88 (0.53 to 1.48)	0.637	77.9
Elective surgery [2,6]	2	537	126/270	119/267	1.05 (0.87 to 1.26)	0.628	0

CI, confidence interval; CKD, chronic kidney disease; CSA-AKI, cardiac surgery-associated acute kidney injury; RR, relative risk; SBIC, sodium bicarbonate.

### Secondary outcomes

**Figure 4:**
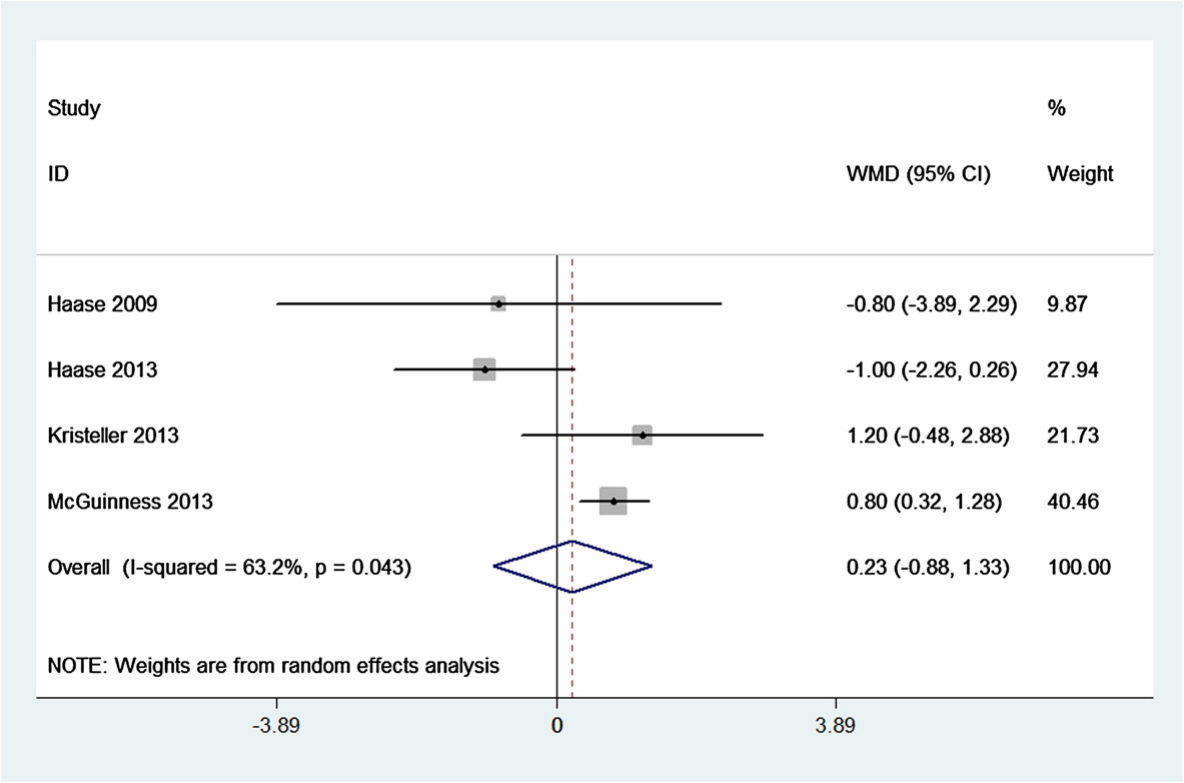
**Forest plot for the meta-analysis of hospital length of stay.** Four randomized controlled trials included [3–6]. CI, confidence interval; WMD, weighted mean difference.

**Figure 5:**
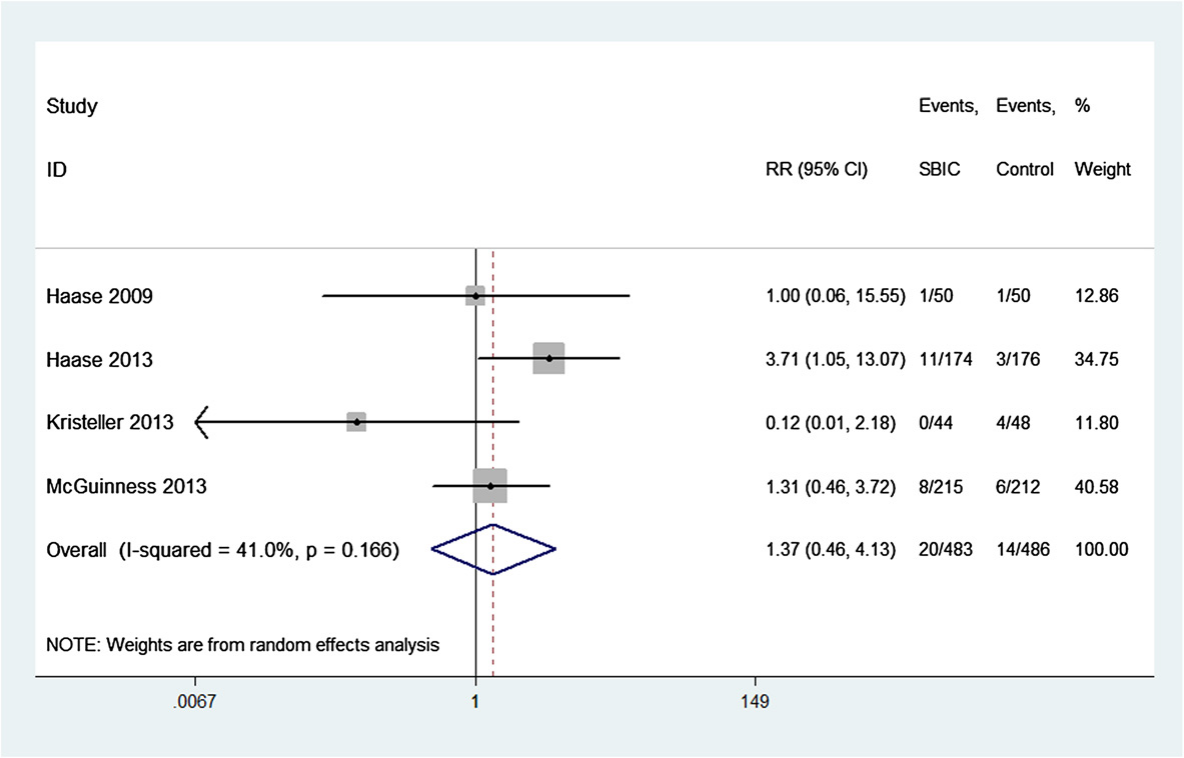
**Forest plot for the meta-analysis of hospital mortality.** Four randomized controlled trials included [3–6]. CI, confidence interval; RR, relative risk; SBIC, sodium bicarbonate.

**Figure 6:**
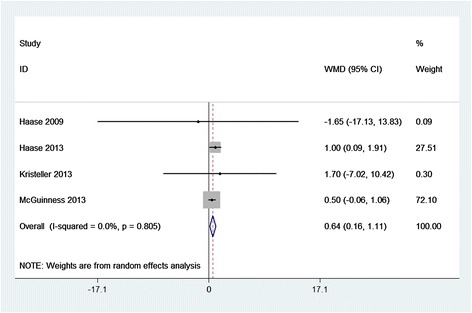
**Forest plot for the meta-analysis of the duration of ventilation.** Four randomized controlled trials included [3–6]. CI, confidence interval; WMD, weighted mean difference.

**Figure 7:**
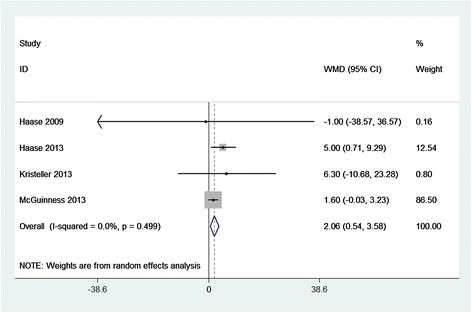
**Forest plot for the meta-analysis of ICU length of stay.** Four randomized controlled trials included [3–6]. CI, confidence interval; WMD, weighted mean difference.

Compared with placebo, SBIC did not alter the clinical outcomes of HLOS (WMD = 0.23 days; 95% CI = −0.88 to 1.33 days; *P* = 0.688; Figure 4), hospital mortality (RR = 1.37; 95% CI = 0.46 to 4.13; *P* = 0.572; Figure 5), RRT (RR = 0.94; 95% CI = 0.49 to 1.82; *P* = 0.861; Additional file 2), or POAF (RR = 1.02; 95% CI = 0.65 to 1.61; *P* = 0.915; Additional file 3). However, SBIC was associated with significant increases in the duration of ventilation (WMD = 0.64 hours; 95% CI = 0.16 to 1.11 hours; *P* = 0.008; Figure 6), ICU length of stay (WMD = 2.06 days; 95% CI = 0.54 to 3.58 days; *P* = 0.008; Figure 7), and incidence of alkalemia (RR = 2.21; 95% CI = 1.42 to 3.42; *P* < 0.001; Additional file 4).

## Discussion

Our meta-analysis clearly suggests that SBIC failed to reduce the incidence of CSA-AKI in adults undergoing cardiac surgery, and the results remained stable in sensitivity analyses. SBIC could not alter the clinical outcomes of RRT, hospital mortality, POAF, and HLOS. Even worse, SBIC was associated with significant increases in the duration of ventilation, ICU length of stay, and incidence of alkalemia.

Three relevant studies that were not eligible for the meta-analysis are summarized in Additional file 5. Among these, two non-RCTs reported that no difference was found in the incidence of CSA-AKI between the SBIC group and the placebo group (3.07% vs. 2.63% and 4.05% vs. 4.14% respectively) [23,24]. Since they were not RCTs (prospective cohort study [23], retrospective cohort study [24]), recall bias, interviewer bias, and selection bias were always a concern. Another study, only enrolling 30 patients with preoperative chronic renal failure, was published as a conference abstract [25] and concluded that SBIC could prevent CSA-AKI. As far as we know, this was the only study that supported the promising result first found by Haase and colleagues [3]. However, the later results of two multicenter trials – using similar patient demographics and treatment as the pilot study [3] – demonstrated that SBIC could not decrease the incidence of CSA-AKI [4,6]. The notable difference was that the pilot study was a single-center trial involving only 100 patients, and a smaller number of participants increased the possibility that chance accounted for the results. Hence, it seemed that the observed benefit in the pilot study and the conference abstract was due to type I error.

Several studies analyzing AKI according to classification and staging systems shared the same conclusion that SBIC could not decrease the incidence of CSA-AKI [4–6,23]. Moreover, a trial involving 350 patients found that SBIC was associated with a significant increase of CSA-AKI in intention-to-treat analysis (odds ratio = 1.60; 95% CI = 1.04 to 2.45) [4]. Consistent with these clinical endpoints, SBIC did not decrease sCr and even increased sCr and neutrophil gelatinase-associated lipocalin [4,5]. Furthermore, patients infused with SBIC needed more noradrenaline, crystalline, and colloidal fluids [23].

The pathophysiology mechanisms of CSA-AKI included ischemia and reperfusion injury, oxidative stress, inflammation, and free hemoglobin [7,26]. Cardiopulmonary bypass could cause the release of free hemoglobin [27], which is converted into methemoglobin and precipitates in the distal tubule in an acidic environment, consequently impairing renal function. The acidic environment also facilitates the generation of injurious hydroxyl radicals [17]. Urinary alkalization via SBIC may thus protect the kidney from injuries caused by tubular cast formation and reactive oxygen species [28]. Based on the postulated mechanism, urinary alkalinization theoretically could protect patients at risk of AKI from CSA-AKI. All of the included studies, except the conference abstract [2], demonstrated that the plasma and urinary alkalinization were achieved via intravenous SBIC [3–6,23]. However, why was the reduced incidence of CSA-AKI not realized? One study speculated that the significantly increased power of hydrogen was not adequate enough to alter the clinical outcome of CSA-AKI [23]. Although whether the promising strategy was challenged is unknown, we could conclude – based on the unchanged/increased biomarkers of renal injury, incidence of CSA-AKI, and demand for drugs – that SBIC showed no beneficial effect on the prevention of CSA-AKI.

Our meta-analysis also demonstrated that SBIC could not alter the clinical outcomes of RRT, mortality, POAF, or HLOS; however, SBIC could prolong the duration of ventilation and ICU length of stay and increase the incidence of alkalemia. These results were not conclusive because they were regarded as secondary outcomes both in the original studies and in our analysis. However, the risk of all secondary outcomes except RRT presented increasing trends. This might not happen by chance but may occur because of scientific factors. CSA-AKI was reported to independently correlate with prolonged/increasing ICU length of stay, HLOS, mortality, and medical cost and even minimal increases in sCr were associated with poor prognosis [14]. SBIC could induce a metabolic alkalosis, as was also indicated by our meta-analysis, and consequently lead to prolonged duration of ventilation [5]. Additionally, two studies reported that SBIC could cause decreased arterial blood pressure [4,23], which was sometimes an emergency situation for postoperative patients. Thus, combining findings of primary and secondary outcomes with the biomarkers of AKI, it can be concluded that SBIC shows no benefits on the prevention of CSA-AKI, and even induces potential harms.

Routine use of SBIC should not be recommended for urinary alkalinization to prevent CSA-AKI, according to our findings. Considering the futility and potential harm confirmed by our findings and the early termination of two trials [4,6], larger-scale trials in the same setting are not encouraged. Moreover, SBIC should be administrated with caution in patients undergoing cardiac surgery. However, since urinary alkalization with SBIC for the prevention of CSA-AKI is reasonable and confirmed by experimental research, further studies focusing on patients without risk of postoperative acute renal dysfunction or pre-existing renal impairment might be promising.

Several limitations should be taken into account. Firstly, only five studies were included and one of them was a conference abstract. However, our sensitivity analysis stratified by article type showed that it did not substantially alter the pooled estimate. Moreover, moderate heterogeneity was observed in our meta-analysis. This heterogeneity might be attributed to various definitions of CSA-AKI, sample size, baseline characteristics of patients, study quality, and surgery type. Additionally, the interventions (timing and dose of SBIC and duration of SBIC maintenance) and other adjuvant measures (hemodynamic management, blood transfusion, diuretics, and medications) might also account for the heterogeneity. Despite the present heterogeneity, the pooled estimate remained stable in the sensitivity analysis. Finally, some unpublished and missing data might lead bias to the pooled effect.

## Conclusions

Although various limitations exist, our meta-analysis clearly suggests that SBIC does not reduce the incidence of CSA-AKI and fails to show any beneficial effect on clinical outcomes in adult patients undergoing cardiac surgery. However, SBIC is associated with longer duration of ventilation, prolonged ICU length of stay, and increased risk of alkalemia. There is thus a lack of evidence supporting SBIC for the prevention of CSA-AKI. Moreover, perioperative SBIC infusion in patients undergoing cardiac surgery should be administrated with caution.

## Key messages

- CSA-AKI is a severe and frequent postoperative complication in patients after cardiac surgery, and the effective prophylaxis of CSA-AKI remains to be established.
- SBIC does not reduce the incidence of CSA-AKI and fails to benefit the clinical outcomes of RRT, hospital mortality, POAF, and HLOS. SBIC could significantly increase duration of ventilation, ICU length of stay, and incidence of alkalemia.
- There is a lack of evidence supporting SBIC for the prevention of CSA-AKI.

## Additional files

Additional file 1:
**is a table presenting the outcomes of the five RCTs included in the meta-analysis.**
Additional file 2:
**shows a Forest plot for the meta-analysis of the incidence of RRT.**
Additional file 3:
**shows a Forest plot for the meta-analysis of the incidence of POAF.**
Additional file 4:
**shows a Forest plot for the meta-analysis of the incidence of alkalemia.**
Additional file 5:
**is a table presenting characteristics of the three relevant studies included in the systematic review.**

## Abbreviations

AKI: acute kidney injury
CI: confidence interval
CSA-AKI: cardiac surgery-associated acute kidney injury
HLOS: hospital length of stay
POAF: postoperative atrial fibrillation
RCT: randomized controlled trial
RR: relative risk
RRT: renal replacement therapy
SBIC: sodium bicarbonate
sCr: serum creatinine
WMD: weighted mean difference
