# Supplementary material for: Sodium bicarbonate in the prevention of cardiac surgery-associated acute kidney injury: a systematic review and meta-analysis
Source: Crit Care. 2014 Sep 12;18(5):517. doi: 10.1186/s13054-014-0517-x (PMC4177432; doi:10.1186/s13054-014-0517-x)
Supplement: Additional file 1: — is a table presenting the outcomes of the five RCTs included in the meta-analysis. [file 13054_2014_517_MOESM1_ESM.doc]

| Table S1 The outcomes of the five included RCTs in the meta-analysis | | | | | | | | | |
| --- | --- | --- | --- | --- | --- | --- | --- | --- | --- |
| Study ID | primary outcome |  | Secondary outcomes | | | | | | |
|  | CSA-AKI |  | Duration of ventilation (h) | ICU length of stay (h) | HLOS  (d) | Hospital mortality | RRT | POAF | alkalemia |
| Haase et al 2009 [3] | 16/50  vs. 26**/**50 |  | 27.62±35.25  vs. 29.27±43.33 | 73.80±98.70  vs. 74.80±92.90 | 10.40±7.20  vs. 11.20±8.50 | 1/50  vs. 1/50 | 2/50  vs. 2/50 | 5/50  vs. 4/50 | 3/50  vs. 0/50 |
| Del Duca et al 2010 [2] | 26/55  vs. 26/55 |  | NA | NA | NA | NA | NA | NA | NA |
| Haase et al 2013 [4] | 83/174  vs. 64/176 |  | 18.00±4.33  vs. 17.00±4.33 | 33.00±24.33  vs. 28.00±15.67 | 17.00±6.00  vs. 18.00±6.00 | 11/174  vs. 3/176 | 8/174  vs. 6/176 | 21/174  vs. 20/176 | 37/174  vs. 13/176 |
| Kristeller et al 2013 [5] | 14/44  vs. 20/48 |  | 9.60±27.90  vs. 7.90±10.00 | 53.90±50.3  vs. 47.60±29.00 | 7.50±5.20  vs. 6.30±2.40 | 0/44  vs. 4/48 | NA | 7/44  vs. 9/48 | 35/44  vs. 21/48 |
| McGuinness et al 2013 [6] | 100/215  vs. 93/212 |  | 12.70±3.33  vs. 12.20±2.53 | 27.80±8.73  vs. 26.20±8.50 | 10.00±2.70  vs. 9.20±2.40 | 8/215  vs. 6/212 | 7/215  vs. 10/212 | NA | NA |
| RRT= renal replacement therapy; ICU= intensive care unit; HLOS=hospital length of stay; POAF=postoperative atrial fibrillation. | | | | | | | | | |
