# Supplementary figures and images for: Sodium bicarbonate in the prevention of cardiac surgery-associated acute kidney injury: a systematic review and meta-analysis
Source: Crit Care. 2014 Sep 12;18(5):517. doi: 10.1186/s13054-014-0517-x (PMC4177432; doi:10.1186/s13054-014-0517-x)

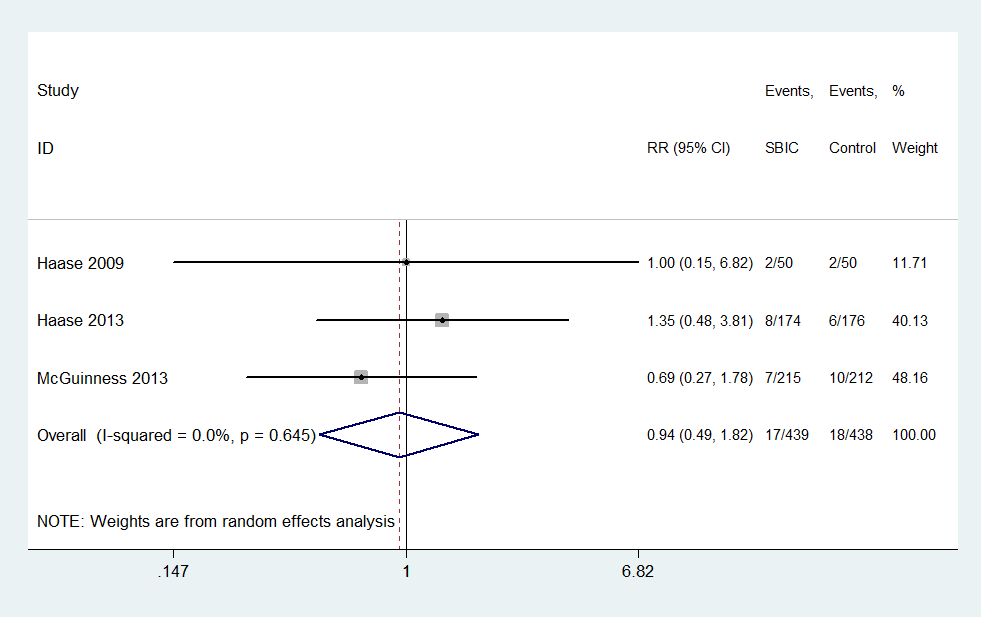

Supplement: Additional file 2: — shows a Forest plot for the meta-analysis of the incidence of RRT. [file 13054_2014_517_MOESM2_ESM.tiff]

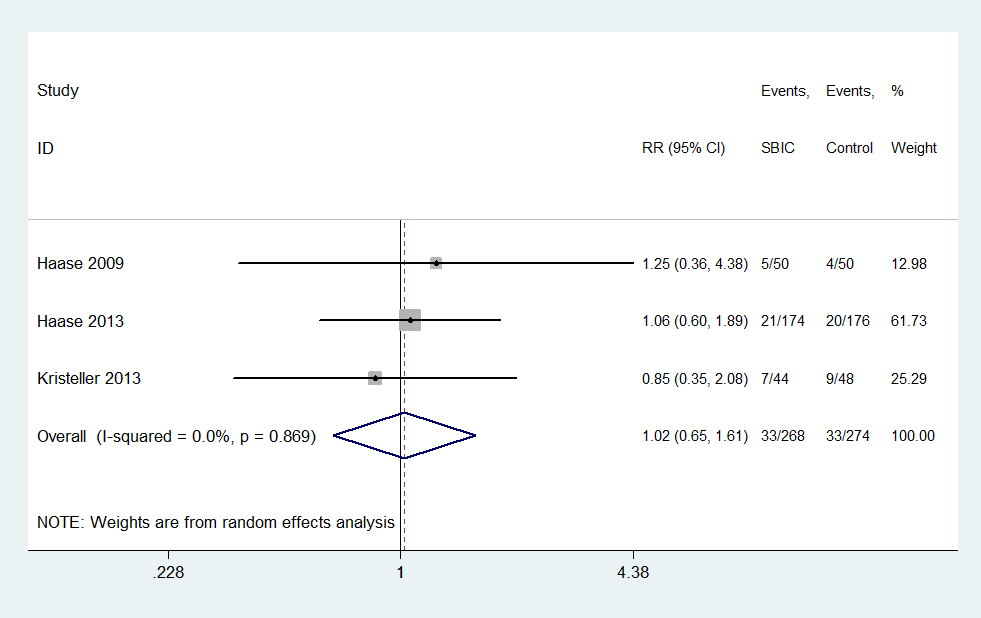

Supplement: Additional file 3: — shows a Forest plot for the meta-analysis of the incidence of POAF. [file 13054_2014_517_MOESM3_ESM.tiff]

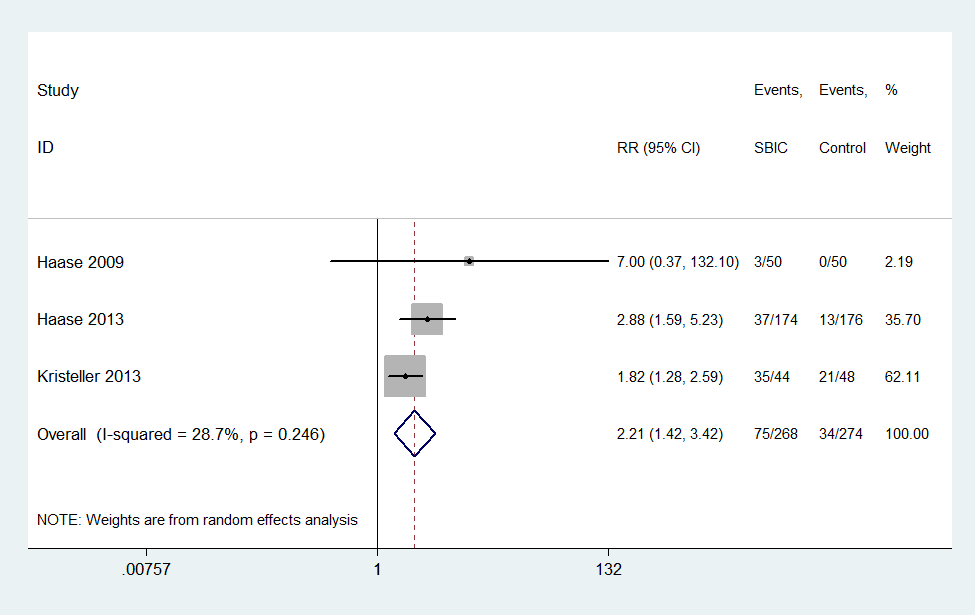

Supplement: Additional file 4: — shows a Forest plot for the meta-analysis of the incidence of alkalemia. [file 13054_2014_517_MOESM4_ESM.tiff]
