# Supplementary material for: Sodium bicarbonate in the prevention of cardiac surgery-associated acute kidney injury: a systematic review and meta-analysis
Source: Crit Care. 2014 Sep 12;18(5):517. doi: 10.1186/s13054-014-0517-x (PMC4177432; doi:10.1186/s13054-014-0517-x)
Supplement: Additional file 5: — is a table presenting characteristics of the three relevant studies included in the systematic review. [file 13054_2014_517_MOESM5_ESM.doc]

| **Table S2 The characteristics of the 3 relevant studies included in the systematic review** | | | | |
| --- | --- | --- | --- | --- |
| **Study ID** | **Study design/ article type** | **Patient characteristics** | **Methods** | **Results** |
|
| Markovic et al 2012 [25] | Randomized, double-blind, controlled trial/ conference abstract | >18 yrs; sCr above 132 μmol/l (CRF); 30 patients undergoing CABG /Valve surgery/ mixed; CPB | Study compared two groups: patients in SBIC group (n=15) received SBIC and in control group (n=15) received NS after induction of anesthesia with a total of 4 ml/kg/24 h. | The incidence of AKI in the SBIC group was significantly lower than that in the placebo group (data not available). Results showed infusion of SBIC could decrease the incidence of CSA-AKI. |
| Heringlake et al 2012 [23] | Prospective cohort study/ journal article | >18 yrs; NA; 584 patient undergoing aortic /valve surgery/CABG; CPB | Study compared two groups: patients in SBIC group (n=280) received SBIC and in control group (n=304) received balanced electrolyte with procedure of 0.5 mmol/kg BW (bolus) over 30 min after induction of anesthesia; continuous IV infusion of 0.15 mmol/Kg BW/h. | No difference in the incidence of AKI between SBIC group and placebo group was observed (3.07% versus 2.63%). Result showed that SBIC failed to decrease the incidence of CSA-AKI |
| Shrestha et al 2011 [24] | Retrospective cohort study/ conference abstract | NA; NA; 229 patients undergoing cardiac surgery; NA | Study compared two groups: patients in SBIC group (n=280) received peri-operative SBIC infusion and in control group (n=304) received nothing. | No difference in the incidence of AKI between SBIC group and placebo group was observed (4.05% versus 4.14%). Result showed that SBIC failed to decrease the incidence of CSA-AKI |
| CABG=coronary artery bypass grafting; NS=normal saline; NA=not available; CRF=chronic renal failure. | | | | |
